# Paper-like graphene-Ag composite films with enhanced mechanical and electrical properties

**DOI:** 10.1186/1556-276X-8-32

**Published:** 2013-01-17

**Authors:** Rungang Gao, Nantao Hu, Zhi Yang, Qirong Zhu, Jing Chai, Yanjie Su, Liying Zhang, Yafei Zhang

**Affiliations:** 1 Key Laboratory for Thin Film and Microfabrication of Ministry of Education, Research Institute of Micro/Nano Science and Technology, Shanghai Jiao Tong University, Shanghai 200240, People's Republic of China; 2 Instrumental Analysis Center, Shanghai Jiao Tong University, Shanghai 200240, People's Republic of China

**Keywords:** Graphene, Ag particles, Composite films, Graphene oxide, *In situ* reduction, Graphene paper, Electrical properties, Mechanical properties

## Abstract

In this paper, we have reported that paper-like graphene-Ag composite films could be prepared by a facile and novel chemical reduction method at a large scale. Using ascorbic acid as a reducing agent, graphene oxide films dipped in Ag^+^ aqueous solutions can be easily reduced along with the decoration of different sizes of Ag particles distributed uniformly. The results reveal that the obtained films exhibit improved mechanical properties with the enhancement of tensile strength and Young's modulus by as high as 82% and 136%, respectively. The electrical properties of graphene-Ag composite films were studied as well, with the sheet resistance of which reaching lower than approximately 600 Ω/□. The graphene-Ag composite films can be expected to find interesting applications in the area of nanoelectronics, sensors, transparent electrodes, supercapacitors, and nanocomposites.

## Background

Graphene, a two-dimensional single atomic layer of *sp*^*2*^-hybridized carbon arranged in a honeycomb structure, has generated tremendous interest due to its unique combination of electronic, mechanical, chemical, and thermal properties [1-4]. Many potential applications in various fields, including filler materials [5,6], field-emission devices [4], nanoscale electronic devices [7], sensors [8-10], transparent electrodes [11-14], and so on [15-18], have been reported.

Large-scale preparation of paper-like graphene films has aroused much attention for their unique mechanical and electrical properties [15,16,19-22]. Some methods, including micromechanical exfoliation [1], chemical vapor deposition [12,23-25], and self-assembly [26-32] have been used to prepare this fascinating structure of the films, which have great potential for the applications in transparent electrodes [25], supercapacitors [33], biosensors [34], etc. Meanwhile, some noble metal nanoparticles have been added into the graphene films to improve the electronic and electrochemical properties of the composite films [31,32] using many methods, such as chemical reduction [33], electrochemical reduction [34], biochemical reduction [35], and *in situ* thermal reduction [36]. As a result, the fabricated composite films have shown a tremendous potential in improving the electrochemical properties [34] and as an electrode of biosensors [35].

Although many efforts and applications have been achieved for these novel carbon films, it is still a great challenge to develop a novel method to prepare the films at a large scale. Herein, we report a new method to prepare graphene-Ag composite films with excellent and improved properties, which are fabricated by the large-scale assembly of graphene oxide films, followed by *in situ* reduction of graphene oxide films together with Ag^+^ by ascorbic acid. The mechanical and electrical properties of the obtained graphene-Ag composite films are also investigated.

## Methods

### Materials

The natural graphite powder (carbon content 99.999%) in the experiment was purchased from Qingdao Tianyuan Carbon Co. Ltd, Qingdao, China. Other solvents and reagents were of analytical reagent grade and used as received.

### Preparation of graphene-Ag composite films

**Figure 1:**
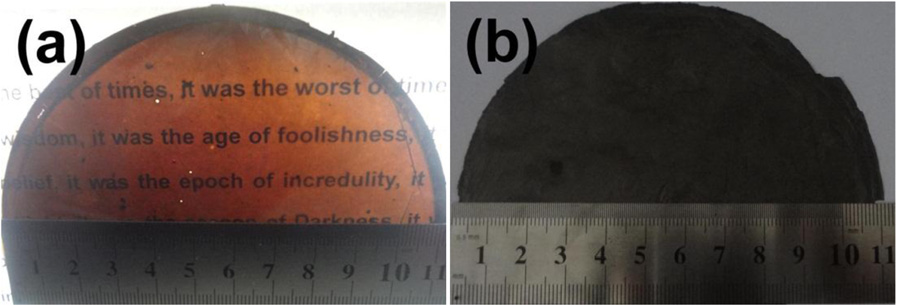
**Photographs of samples.** (**a**) Graphene oxide films and (**b**) graphene-Ag composite films with the amount of 10 mg AgNO_3_.

**Figure 2:**
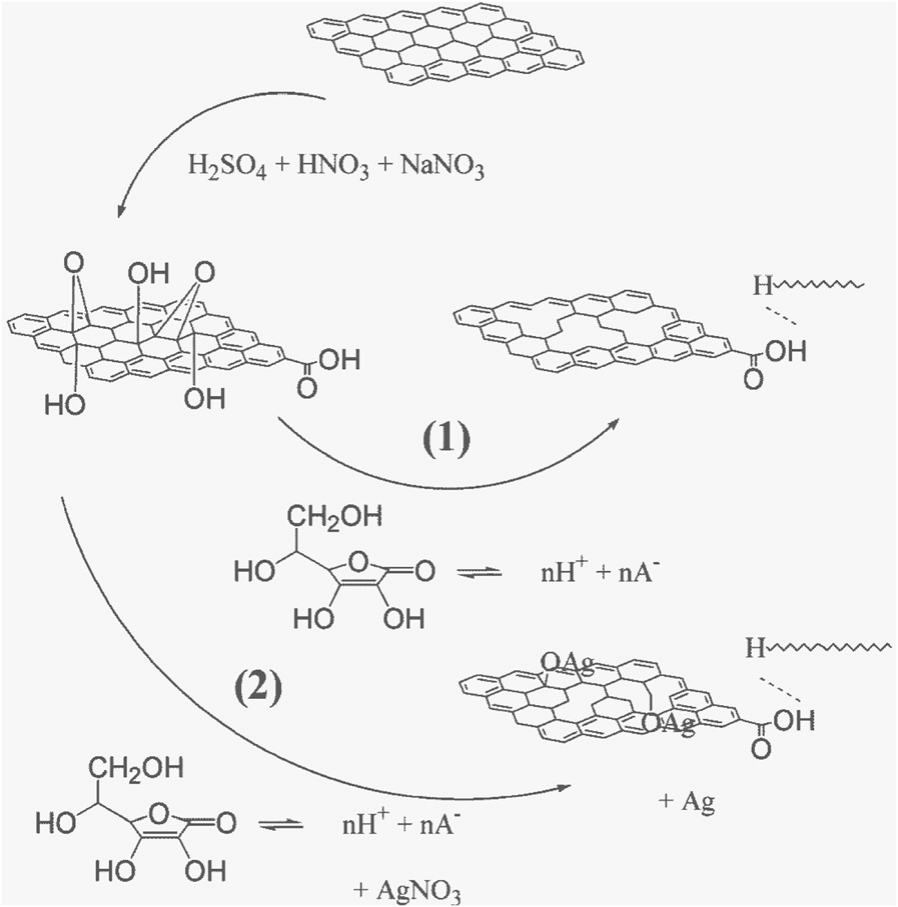
Schematic illustration of the chemical route for the synthesis of graphene-Ag composite films.

Graphene oxide was synthesized through the modified Hummers method [37] as stated in our previous reports [2,18,38]. Prior to reduction, the synthesized graphene oxide (0.15 g) was dispersed in 50 mL of deionized water by ultrasonic treatment (1,000 W, 40 kHz) for 2 h, and then, the yellow-brown dispersion was poured into a polytetrafluoroethylene (PTFE) plate with a diameter of 11.5 cm and heated at 80°C for 24 h. Finally, the brown-black films with a diameter of 10 to 11 cm and thickness of 10 μm could be obtained as shown in Figure 1a. In order to reduce the graphene oxide films, ascorbic acid was used as a reducing agent [38,39]. To obtain graphene films, 150 mg ascorbic acid was dissolved in water, followed by soaking the graphene oxide films into the solution for a certain time in order to determine an optimized period. In addition, to obtain graphene-Ag composite films, 150 mg ascorbic acid was dissolved into the AgNO_3_ aqueous solution (100 mL, 2 to 300 mg), and the graphene oxide films were soaked in the mixed solution for 5 h. The schematic illustration of two chemical synthesis routes is described in Figure 2. After washing with deionized water, the final black paper-like graphene films and graphene-Ag composite films (Figure 1b) were obtained after heated at 80°C for 2 h, respectively.

### Characterizations

Atomic force microscope (AFM) image was taken with the Multimode Nanoscope V scanning probe microscopy system (Veeco Instruments Inc., Plainview, NY, USA) using tapping mode with Picoscan v5.3.3 software. The morphology of the films were observed using a scanning electron microscope (SEM) using a Carl Zeiss ULTRA 55 (Carl Zeiss, Oberkochen, Germany) with energy dispersive X-ray (EDX) spectrometry mode. The crystal structures of the films were examined by X-ray diffraction (XRD; D/MAX-2200, Rigaku, Tokyo, Japan) with Cu Kα (*λ* = 1.5418 Å) and 2*θ* from 5° to 85°. The Raman spectra were obtained using a Senterra R200-L Raman spectrometer (Bruker Optik GmbH, Ettlingen, Germany) with a 514-nm line of laser source. Fourier transform infrared (FTIR) spectra were recorded using a Vertex 70 vacuum FTIR spectrometer (Bruker Optik GmbH) and scanned from 4,000 to 400 cm^−1^ with KBr as background. Thermogravimetric analysis (TGA; Pyris 1, PerkinElmer, Waltham, MA, USA) was performed under a highly pure nitrogen atmosphere with a heating rate of 1°C to 10°C/min from 30°C to 700°C. The films with 5-mm width and 4- to 5-cm length were measured by dynamic mechanical analysis (DMA; TA-Q800, TA Instruments, Newcastle, DE, USA) at the room temperature. A four-probe detector (RTS-8, 4 PROBES TECH, Guangzhou, China) was used to measure the sheet resistance of the films.

## Results and discussion

**Figure 3:**
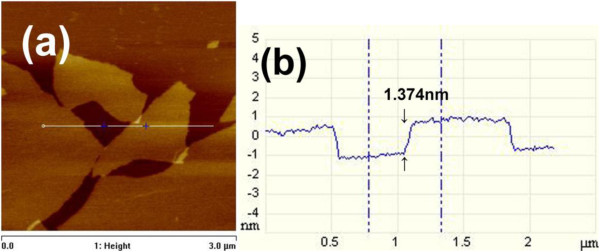
**AFM images of graphene oxide.** (**a**) AFM image and (**b**) the height profile of the image.

**Figure 4:**
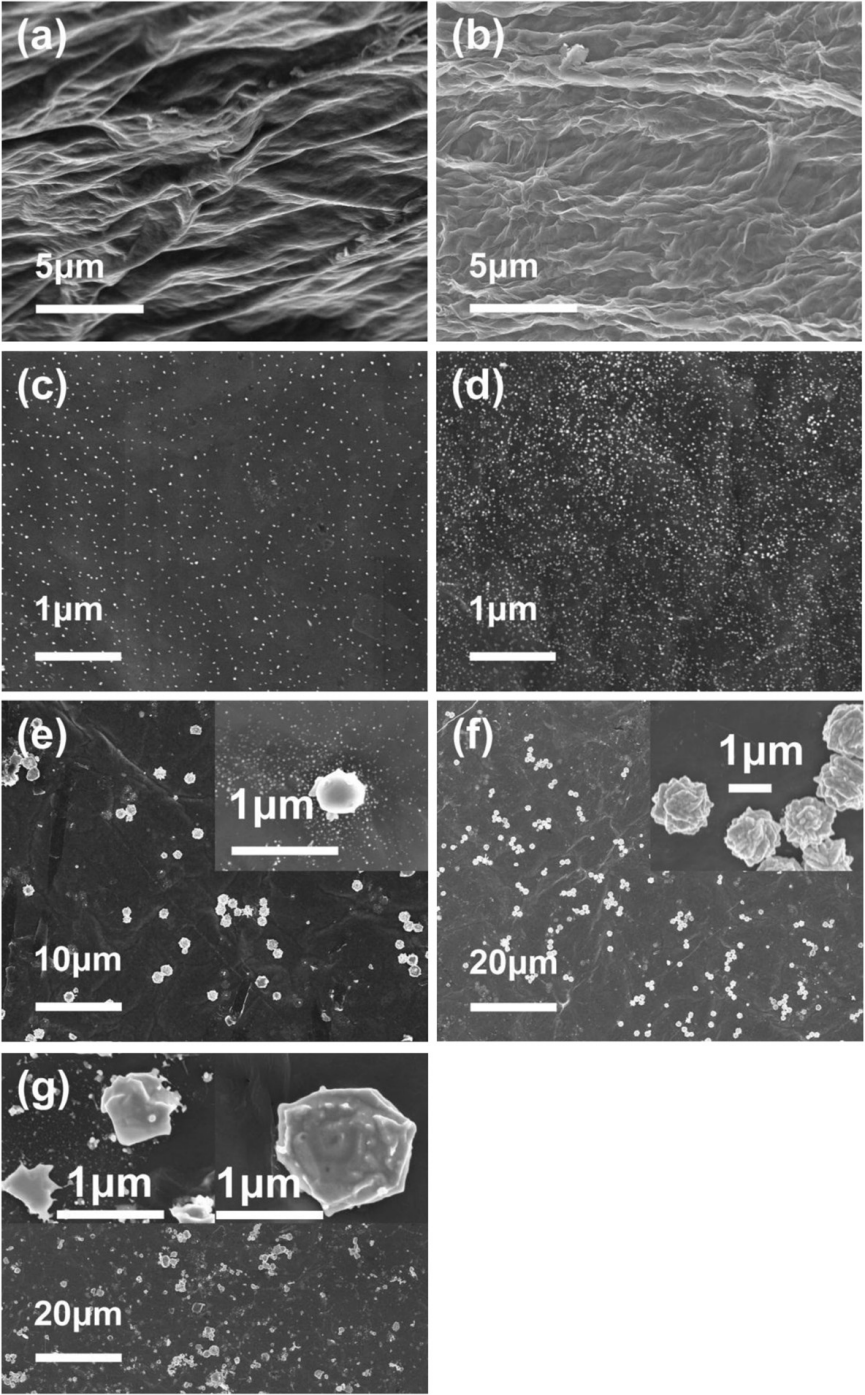
**SEM images of surface morphologies of different films.** (**a**) Graphene oxide films, (**b**) graphene films (reduced by ascorbic acid), and (**c** to **g**) graphene-Ag composite films (the amount of AgNO_3_ is from 2 to 300 mg in each film).

The modified Hummers method had been used to prepare graphene oxide. By sonicating the graphene oxide in water, graphene oxide sheet aqueous solution was obtained. From the tapping-mode AFM image as shown in Figure 3, it is observed that the thickness of the obtained graphene oxide sheet is approximately 1.05 nm, which indicates that the graphene oxide can be easily exfoliated into single layer by the oxidation and sonication treatment [40]. The graphene oxide films with a large area were fabricated by casting method. The graphene oxide sheets can be easily assembled into graphene oxide films by volatizing water in the oven at 80°C. PTFE, a hydrophobic substrate, is used to make sure that the films are easily peeled off and the large-area free-standing films fabricated. As shown in Figure 1a, the yellow-brown paper-like films with a semitransparent characteristic are obtained. In order to obtain the graphene films, ascorbic acid, as an excellent reducing agent, has been used here to reduce the graphene oxide films [39]. As a result of the reduction process, the opaque graphene films with black color (Figure 1b) are obtained. Excitingly, the morphology of the graphene films can be perfectly maintained after the reduction process (Figure 4a,b), which suggests that this facile and novel method is suitable for the large-scale production of graphene films. For the improvement of the conductivity of the films, Ag particles have been *in situ* introduced during the process of the reduction reaction. The morphology of the graphene-Ag composite films has been observed by SEM, as shown in Figure 4. It can be found that the films are decorated with Ag particles with an average particle size from approximately 20 nm to approximately 1 μm (Figure 4c,d,e,f,g). When the mass ratio of AgNO_3_/graphene oxide is 1:75, these Ag particles with a size of about 20 nm were distributed uniformly at the surface of the composite films (Figure 4c). When the mass ratio is increased to 1:30, the Ag particles with the same size as shown in Figure 4c disperse well while the intensity of Ag particles distributed on the surface of the films increase (Figure 4d). As the mass ratio is increased to 1:15, the size of Ag particles is remarkably increased along with partial particles deposited together to form bigger spheres with a diameter of approximately 1 μm (Figure 4e). With the increase of the mass ratio to 1:7.5, Ag particles further aggregate but still disperse well (Figure 4f). Finally, with the mass ratio of 2:1, the morphology of those Ag particles becomes bigger and irregular (Figure 4g).

**Figure 5:**
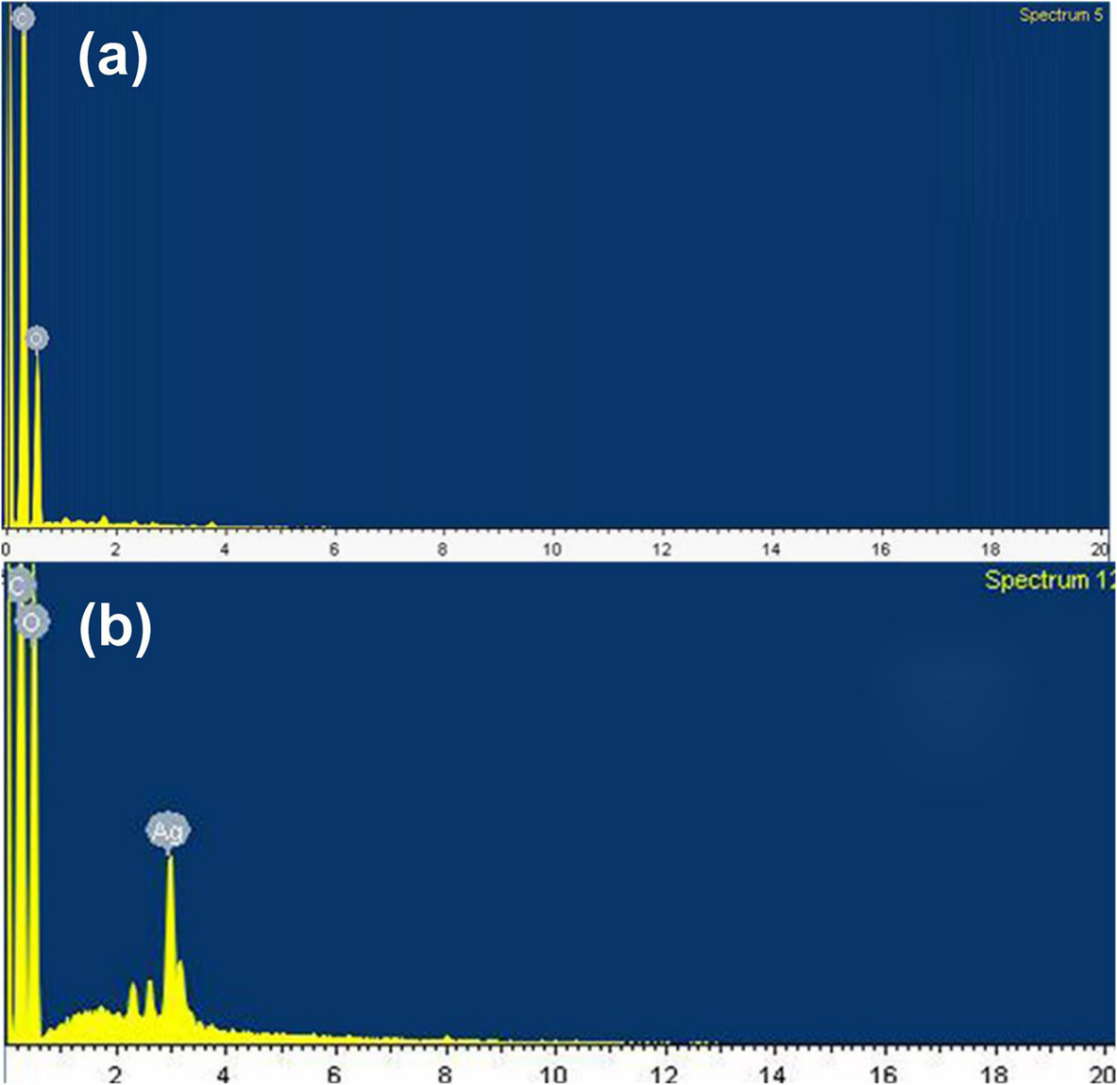
**EDX spectra of graphene and composite films.** (**a**) Graphene films and (**b**) graphene-Ag composite films; the mass ratio of AgNO_3_/graphene oxide is 2:1.

EDX is used to qualitatively determine the variation of relative ratio of each element. The results in Figure 5 and Table 1 show that the atomic ratios of C/O of the graphene films and graphene-Ag composite films are various from 2.2 to 2.5, lower than those in a previous study [11]. Compared with the graphene oxide films (the atomic ratio of C/O is approximately 1.5), the increased atomic ratio of C/O of the composite films means that the reduction progress has occurred. Simultaneously, the weight percentages of the Ag element may influence the reaction in some way. When the amount of AgNO_3_ reaches to 300 mg, the atomic ratio of C/O is far lower, indicating that the reduction process may be affected when the amount of AgNO_3_ is excessive. As for EDX results, the appropriate amount of AgNO_3_ is around 5 to 10 mg.

**Table 1:** Elements of all films measured by EDX

**AgNO**_**3 **_**(mg)**	**Weight (%)**			**Atomic (%)**			**Atomic ratio (C/O)**
	**C**	**O**	**Ag**	**C**	**O**	**Ag**	
GO	50.03	44.03		58.11	39.17		1.48
0	65.57	34.43		71.72	28.28		2.54
2	61.54	37.83	0.63	68.37	31.55	0.08	2.17
5	64.85	34.26	0.89	71.52	28.37	0.11	2.52
10	63.46	34.42	2.12	70.88	28.86	0.26	2.46
20	59.06	35.09	5.85	68.63	30.61	0.76	2.24
300	51.86	40.87	7.27	62.22	36.81	0.97	1.69

0 stands for the graphene film reduced for 5 h.

**Figure 6:**
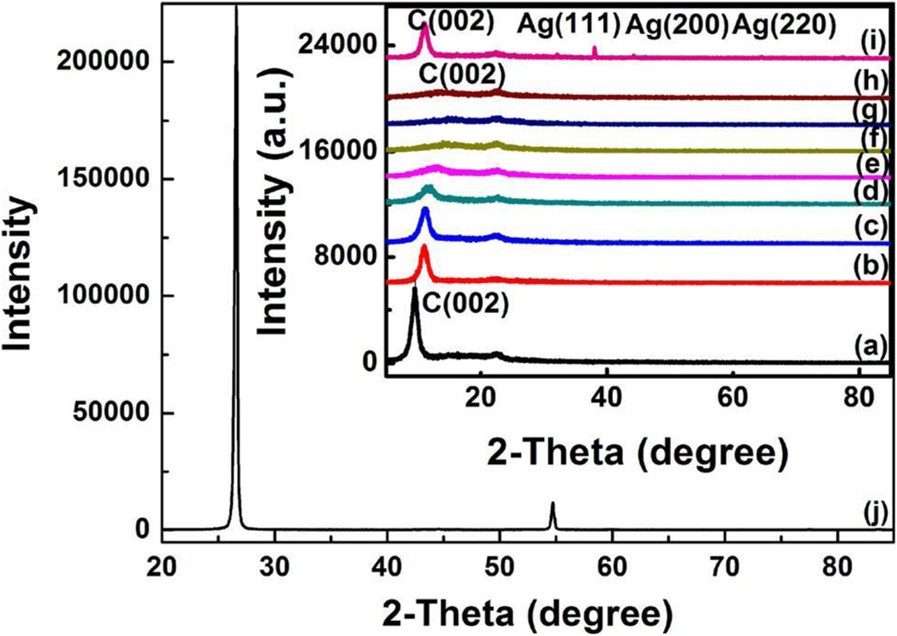
**XRD patterns of graphite, graphene oxide, and graphene-Ag composite films.** (**a**) Graphene oxide films, (**b** to **d**) graphene films (reduced by ascorbic acid), (**e** to **i**) graphene-Ag composite films (the amount of AgNO_3_ was from 2 to 300 mg in each film), and (**j**) graphite.

The XRD patterns also support the results from SEM and EDX. Only when the amount of AgNO_3_ is 300 mg, the final weight percentage of Ag is more than 7%, so the crystal structure and ordering of Ag particles can be characterized by XRD. As shown in Figure 6 (i), the characteristic peaks at 38.02°, 44.24°, and 64.56° correspond with the (111), (200), and (220) planes of the cubic Ag crystal (JCPDS no. 04–0783), respectively, which indicates that the metallic Ag particles are formed after being reduced. According to the Bragg spacing equation, the characteristic peak of carbon (002) changes from 26.6° (Figure 6 (j), pristine graphite powder) to 9.6° (Figure 6 (a), graphene oxide) and sharply weakens, showing that the layer-to-layer distance (d-spacing) from 0.67 to 1.84 nm, which means the structure of *sp*^*2*^ carbon has been deformed after the introduction of some oxygen functional groups. At the same time, the layer-to-layer distance of the graphene-Ag composite films has also been changed from 1.20 to 1.61 nm, indicating that some of the oxygen functional groups have been reduced. As shown, the graphene-Ag composite films have a shorter distance from 1.20 to 1.34 nm than the graphene films from 1.56 to 1.61 nm, suggesting that AgNO_3_ is beneficial to the reduction process and the suitable amount of AgNO_3_ is 10 mg.

**Figure 7:**
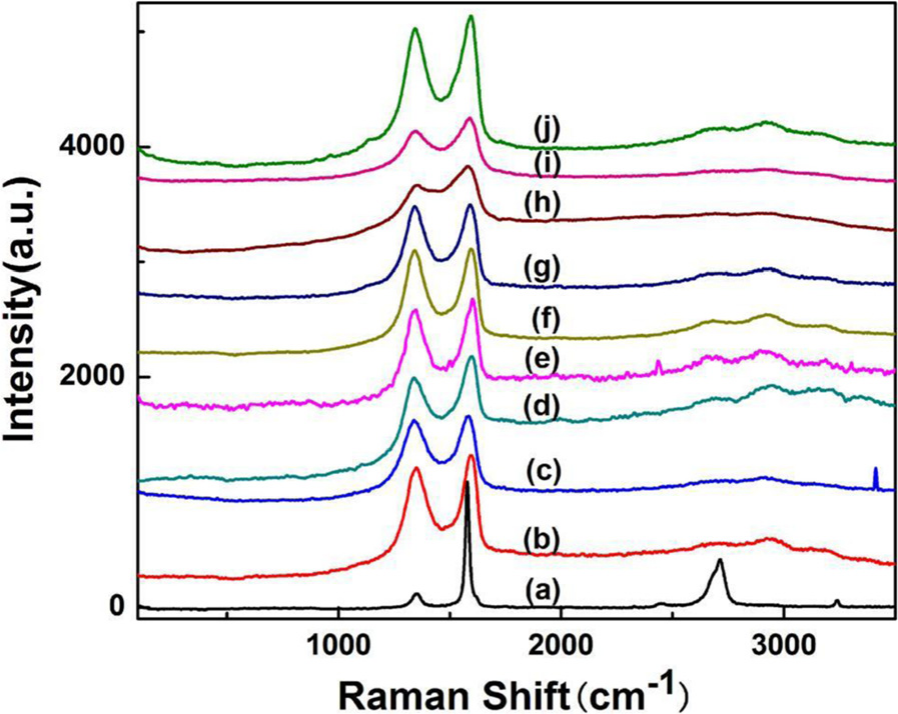
**Raman spectra of graphite, graphene oxide, and graphene-Ag composite films.** (**a**) Graphite, (**b**) graphene oxide film, (**c** to **e**) graphene films (reduced by ascorbic acid), and (**f** to **j**) graphene-Ag composite films (the amount of AgNO_3_ was from 2 to 300 mg in each film).

The Raman scattering signals were measured on the graphite powder (Figure 7 (a)), graphene oxide films (Figure 7 (b)), the graphene films (Figure 7 (c to e)), and the graphene-Ag composite films (Figure 7 (f to j)). The Raman spectra exhibit two main characteristic peaks, the D band (approximately 1,345 cm^−1^) and G band (approximately 1,590 cm^−1^). The G band represents the plane vibrations with *E*_2g_ symmetry and is mainly sensitive to the configuration of *sp*^*2*^ sites, while the D band is related to the breathing mode of *κ*-point phonon of *A*_1g_ symmetry. As the graphite was oxidized into graphene oxide, the G band is broadened and the D band increases substantially, indicating the decrease in size of the in-plane *sp*^*2*^ sites, possibly because of the extensive oxidation and ultrasonic exfoliation [41]. When graphene oxide is reduced by ascorbic acid for 5 h, the increase of the *I*_D_/*I*_G_ intensity ratio of graphene is observed compared to that of the graphene oxide. Finally, when AgNO_3_ was used, the increase of D band also occurred. This change suggests an increase in the average size of the *sp*^*2*^ sites upon reduction of graphene oxide, which indicates that the reduction reaction has taken place and agrees well with the Raman spectrum of the graphene oxide reduced by hydrazine as reported by Stankovich et al. [42].

**Figure 8:**
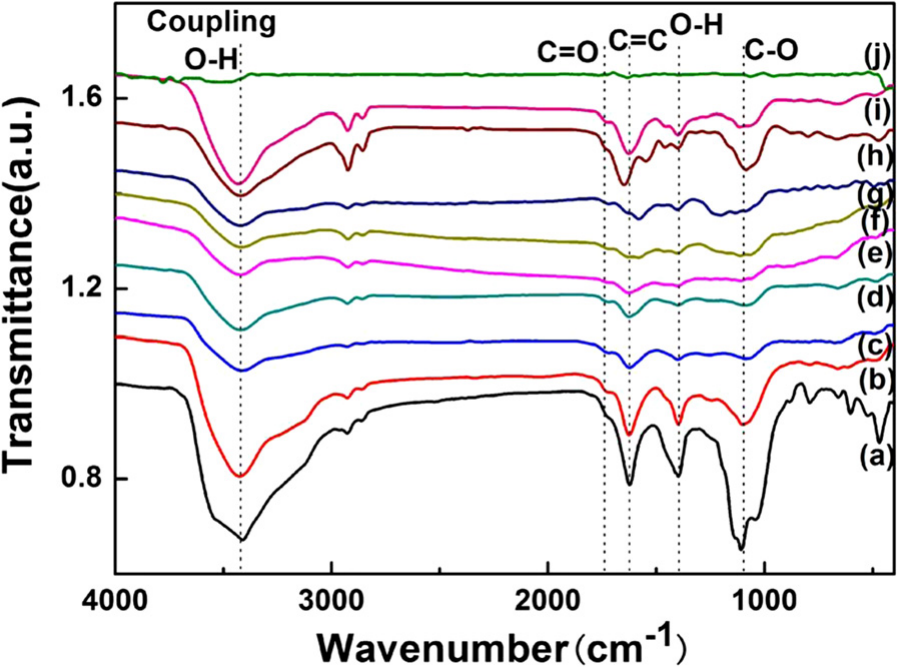
**FTIR spectra of graphite, graphene oxide, and graphene-Ag composite films.** (**a**) Graphene oxide films, (**b** to **d**) graphene films (reduced by ascorbic acid), (**e** to **i**) graphene-Ag composite films (the amount of AgNO_3_ was from 2 to 300 mg in each film), and (**j**) graphite.

FTIR is used to characterize the functional groups in the films shown in Figure 8. When the pristine graphite powder (sample j) is oxidized, many functional groups can be introduced in the graphene oxide films (sample a), which have a peak at approximately 3,410 cm^−1^ arising from the -OH stretching vibrations and peak at approximately 1,730 cm^−1^ of carboxyl C=O, approximately 1,620 cm^−1^ of C-C groups, approximately 1,400 cm^−1^ of O-H, and 1,100 cm^−1^ of alkoxy C-O. After the reduction treatment, most of the absorption peaks are weakened or disappeared, mainly leaving the C-C vibration peaks [11,16]. As far as samples b to d with the reduction time of 1 h (as shown in Figure 8 (b)) are concerned, the peaks remain almost as strong as that of sample a, suggesting that the reduction of sample b has not completely occurred. Meanwhile, the peaks of samples c and d do not have a significant difference, indicating that the period time of 5 h is enough to reduce the graphene oxide. When the amount of AgNO_3_ is added from 2 to 10 mg (samples e to g), the peaks seem to be similar with those of samples c and d since a few existing Ag particles do not block the reaction. However, when the amount of AgNO_3_ is excessive as 20 mg (sample h) and 300 mg (sample i), all peaks become stronger again, which means that the side effects will arise gradually as the amount of AgNO_3_ increases.

**Figure 9:**
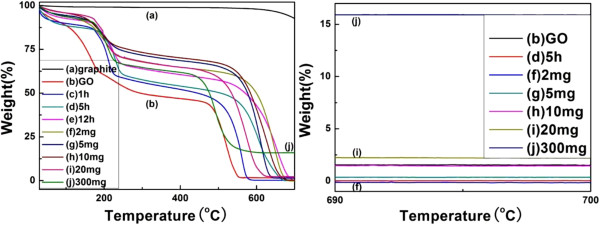
**TGA spectra of graphite, graphene oxide, and graphene-Ag composite films.** (**a**) Graphite, (**b**) graphene oxide film, (**c** to **e**) graphene films (reduced by ascorbic acid), and (**f** to **j**) graphene-Ag composite films (the amount of AgNO_3_ was from 2 to 300 mg in each film).

Thermogravimetric analysis has also been performed. Figure 9 exhibits TGA curves of (a) graphite; (b) graphene oxide; (c to e) graphene films reduced for 1, 5, and 12 h; and (f to j) graphene-Ag composite films with the amount of AgNO_3_ from 2 to 300 mg under nitrogen atmosphere. In the left image of Figure 9, graphene oxide (Figure 9 (b)) and the graphene reduced for only 1 h (Figure 9c) have an inferior thermal stability, while the pristine graphite is quite stable below 600°C. The decomposition of graphene oxide begins at 200°C, which is probably due to the loss of the acidic functional groups and residues. When reduction time is more than 5 h (Figure 9 (d) and (e)), the TGA curves of graphene only exhibit a slight mass loss at a temperature lower than 600°C, which suggests that the enhancement of thermal stability is achieved after the oxygen-containing functional groups are removed during reduction [18,28]. In addition, Ag particles can also affect the thermal stability of graphene. If the amount of AgNO_3_ is appropriate (no more than 10 mg), the TGA curves of graphene-Ag composite films exhibited a mass loss at a temperature lower than 600°C, slightly lesser than that of graphene reduced only by ascorbic acid. However, when the amount of AgNO_3_ is 20 mg and 300 mg, the TGA curves of the composite films turned out to have the same trend as that of graphene oxide. The right image of Figure 9 exhibits the weight loss of partial samples at a temperature from 690°C to 700°C; it can be seen that the residue weight increases as the amount of AgNO_3_ is increased, and more than 15% weight is left at 690°C as the AgNO_3_ is excessive up to 300 mg. We can also find that the residue weight of samples i and j has a little difference with the EDX results. It may be due to the excessive Ag particles which aggregated and deposited nonuniformly on the surface of the graphene-Ag composite films.

**Figure 10:**
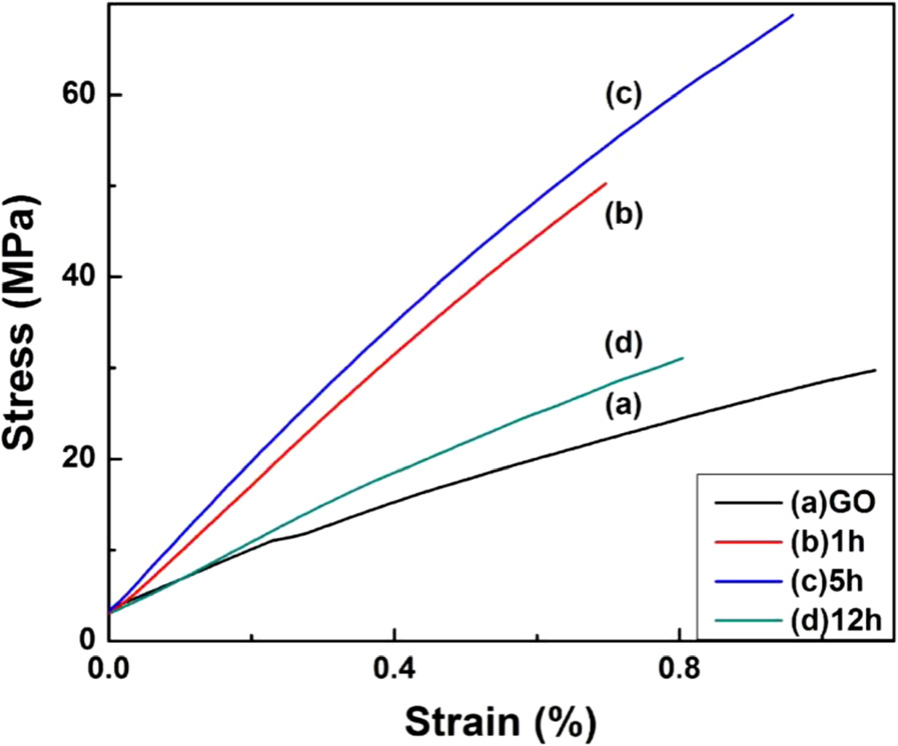
**Mechanical curves of the films tested by DMA.** (**a**) Graphene oxide films and (**b** to **d**) graphene film (reduced by ascorbic acid).

The mechanical properties of graphene oxide films and graphene films have also been studied, as shown in Figure 10 and Table 2. Compared with graphene oxide films, graphene films exhibit enhanced mechanical behaviors. After being reduced for 5 h, the stress of the obtained graphene films increases from 33 to 60 MPa (increased by 82%), and the strain decreases from 1.3% to 0.9%. The preliminary results, a considerable improvement in the Young's modulus of graphene films increased by 136% (up to 7.8 MPa), are encouraging. From Table 2, it can be also observed that the optimal reduction period for the preparation of graphene films is 5 h. Moreover, after Ag particles are decorated, there is little change in the mechanical properties of graphene-Ag composite films compared with the corresponding graphene films.

**Table 2:** Mechanical properties of graphene oxide films and graphene films reduced for different times

**Sample**	**Strain (%)**	**Stress (MPa)**	**Modulus (GPa)**
(a) GO	1.3 ± 0.2	33.0 ± 1.3	3.3 ± 0.3
(b) 1 h	0.8 ± 0.1	49.3 ± 0.9	6.8 ± 0.1
(c) 5 h	0.9 ± 0.1	60.2 ± 0.6	7.8 ± 0.1
(d) 12 h	0.9 ± 0.1	32.5 ± 1.4	3.9 ± 0.2

**Figure 11:**
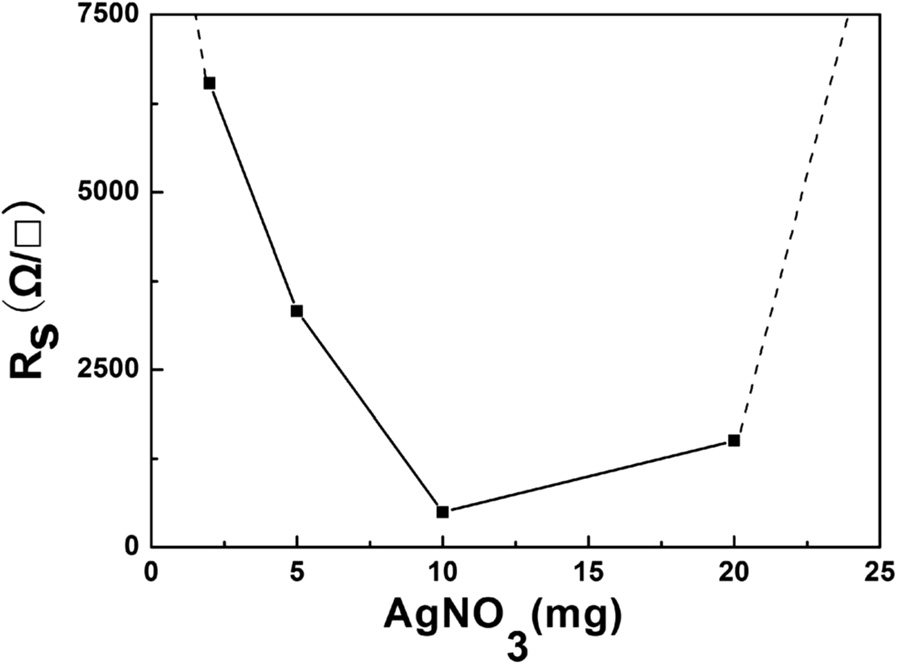
The electrical properties of the graphene-Ag composite films.

Finally, the sheet resistance of these films was measured using the four-probe detector as shown in Figure 11. The electrical properties can be tuned by the addition of a given amount of Ag particles. When the amount of AgNO_3_ is no more than 10 mg, the sheet resistance decreases; on the other hand, when the amount of AgNO_3_ is 20 mg, the sheet resistance increases. When the optimal amount of AgNO_3_ is 10 mg, a minimum sheet resistance of approximately 600 Ω/□ for graphene-Ag composite films is obtained. It can be found that the conductivity of the resultant graphene-Ag composite films can be improved greatly via the uniform decoration of Ag particles.

## Conclusions

In summary, we have demonstrated that graphene-Ag composite films are fabricated in a large scale using a facile chemical reduction method. The graphene oxide sheets can be easily assembled to form free-standing graphene oxide films during the volatilization process on PTFE hydrophobic substrate. After dipping the graphene oxide films into the Ag^+^ aqueous solution, Ag particles can be uniformly distributed on the surface of graphene films using ascorbic acid as a reducing agent. The morphology of the composite films can be maintained during the reduction process. The obtained films have been characterized by AFM, SEM, XRD, Raman, FTIR, TGA, DMA, and a four-probe detector. The results show that the obtained films exhibit improved mechanical properties with the enhancement of tensile strength and Young's modulus by as high as 82% and 136%, respectively. The electrical properties of the graphene-Ag composite films were studied as well, with the sheet resistance of which reaching lower than approximately 600 Ω/□. The composite films hold a great potential for applications in the fields of nanoelectronics, sensors, transparent electrodes, supercapacitors, and nanocomposites.

## Competing interests

The authors declare that they have no competing interests.

## Authors’ contributions

RGG carried out the sample preparation, participated on its analysis, performed all the analyses except AFM and FTIR analyses, and wrote the paper. NTH also wrote the paper and analyzed the samples. JC performed the FTIR analysis. QRZ participated on the AFM analysis and proof corrections. ZY, YJS, LYZ, and YFZ participated in the study guidance and paper correction. All authors read and approved the final manuscript.
